# Enhanced but not superior: Performance outcomes from the inaugural enhanced games

**DOI:** 10.14814/phy2.71123

**Published:** 2026-09-29

**Authors:** Rafael A. Azevedo, Luis F. Coutinho, Monique C. Gonçalves, Higor A. Oliveira, Guilherme J. Gonçalves, Marcus Campos, Hamilton Roschel, Bruno Gualano

**Affiliations:** ^1^ Applied Physiology and Nutrition Research Group, School of Physical Education and Sport, Faculdade de Medicina FMUSP Universidade de São Paulo São Paulo Brazil; ^2^ Center of Lifestyle Medicine, Faculdade de Medicina FMUSP Universidade de São Paulo São Paulo Brazil; ^3^ Faculty of Medical Sciences of Minas Gerais (FC MMG) Belo Horizonte Brazil; ^4^ School of Applied Sciences (FCA) University of Campinas (UNICAMP) Limeira Brazil

**Keywords:** athletic performance, doping, sports performance

## Abstract

The Enhanced Games (TEG) emerged as an athletic event engineered to profit from biotechnological enhancement, such as performance‐enhancing drugs (PEDs) utilization, relying on three premises: shattering world records (WR), enabling athletes to surpass their personal bests (PB), and utilizing PEDs to offset age‐related physical decline. The effectiveness of TEG's PED protocols in achieving these three premises was evaluated. This is a retrospective analysis from 42 TEG athletes across Track, Swimming, and Weightlifting. TEG's performances were compared to current WR and athletes' PB previous to TEG. The anti‐aging PED effects were evaluated by the relationship between the age and performance gaps from PB to TEG competition. Athletes' previous PBs were −5.92% ± 4.68% worse than current WR, which further deteriorated to −8.91% ± 7.52% after TEG, and a single WR was broken. Whereas 19 new PBs were recorded, TEG performances were on average −2.86% ± 7.39% worse than previous PB. Some competitive results were achieved by non‐enhanced athletes. Performance decline continued linearly as the time elapsed since the athletes' physical prime (i.e., age at PB). TEG failed to deliver on core promises of widespread WR and broad athletic enhancement, and PED protocol did not attenuate expected age‐related performance declines.

## Graphical abstract

Overall study design and main findings from The Enhanced Games performances analysis.

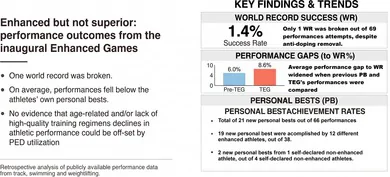

## 1 INTRODUCTION

The Enhanced Games (TEG) (Boardley, 2026) was a showcase athletic event held in Las Vegas on May 24th of 2026, which allowed performance enhancing drugs (PEDs) (Watson et al., 2022) and ruled out any anti‐doping regulation. Fundamentally, TEG was a materialization of a broader business model, ultimately focused on profiting from the massification of PED prescription. From TEG's perspective, the integration of modern medicine and biotechnology to extend human physical capabilities is a natural and desirable evolution in sport and society (Sandbakk & Loland, 2026). This claim is based on the premise that the human body is an incomplete project, which needs constant improvement through biotechnology advances (Cox & Khan, 2025). Thus, individuals are encouraged to treat bodies as ongoing projects requiring constant management, attention, and enhancement, whether through hormone therapy, supplements, fitness regimens, or anti‐aging treatments (Sandbakk & Loland, 2026).

A cornerstone from TEG's business model success is the achievement of world records (WR) by their “enhanced athletes,” which would, theoretically, increase visibility to PED prescription and TEG's profit. Briefly, sports records, such as WR marks, are analytic measurements in exact mathematical–physical entities such as meters, seconds, or kilograms within a standardized spatial–temporal framework defined by sports constitutive rules (Loland, 2001). The decision of TEG to remove from their program other athletic events such as Gymnastics and Boxing could be considered a strategic move to increase their market share through WR. In addition, TEG's PED protocols promise to deliver “the best version of yourself,” ultimately represented by setting new personal best (PB) physical and athletic performances. Based on these premises, TEG's protocols of PEDs would be expected to be effective, since documented examples of enhanced athletic performance under these substances are numerous. For example, between the 1970s and 1980s, there was a state‐sponsored PED prescription system from the German Democratic Republic which systematically administered these substances to thousands of elite athletes. Indeed, this strategy resulted in roughly 150 Olympic medals (Sandbakk & Loland, 2026). More recently, successful athletes and teams have also been caught by anti‐doping agencies, such as BALCO, Lance Armstrong, and/or the Russian doping scheme in Sochi, which reinforce the notion that the positive effects of PEDs on physical performance were worth the high risk for these examples. In this context, some types of PEDs are more commonly used by elite doped athletes, such as androgenic anabolic steroids (AAS), growth hormone (GH), and/or recombinant human erythropoietin (rHuEPO) (Warrier et al., 2024). For example, AAS and/or GH administration has been consistently shown to stimulate acute muscle protein synthesis, leading to chronic increases in lean body mass and maximal strength (Warrier et al., 2024). Similarly, rHuEPO use demonstrates clear physiological benefits, such as elevated hemoglobin mass, increased oxygen‐carrying capacity, and subsequent improvements in maximal oxygen uptake, which translate to better aerobic performance in laboratory settings (Warrier et al., 2024). However, although many PEDs have well‐established physiological and mechanistic effects, the extent to which these adaptations translate into superior competitive outcomes remains dependent on multiple factors beyond pharmacological enhancement alone. For instance, an umbrella review has shown that whereas PEDs such as AAS and GH unequivocally increase lean body mass and muscle strength, there is insufficient evidence directly linking them to improved on‐field sports performance (Warrier et al., 2024). The same scenario occurs for rHuEPO; while it reliably improves maximal aerobic capacity, there is a lack of robust evidence translating this to faster running, cycling, or swimming times in elite competition (Warrier et al., 2024).

In addition to claims regarding athletic enhancement, TEG‐associated initiatives have promoted the use of PEDs as a strategy to attenuate age‐related declines in physical performance and support longevity (Cox & Khan, 2025). This claim is particularly relevant because many athletes participating in the inaugural TEG were older than when they achieved their PB. Consequently, the event provides a unique opportunity to examine whether the athletic outcomes observed are consistent with the proposition that pharmacological enhancement can meaningfully offset age‐related declines in performance.

Therefore, the aims of the present study were to evaluate whether the inaugural TEG's performance‐enhancing protocols fulfilled their three core promises: (i) generating new WR; (ii) enabling athletes to exceed their previous PB; and (iii) attenuating the expected age‐related declines in athletic performance.

## 2 MATERIALS AND METHODS

### 2.1 Sample and data extraction

This study is a retrospective analysis utilizing publicly available, secondary data obtained from official websites and open registries. In accordance with institutional and national guidelines for observational research relying exclusively on public domain data without direct human contact or intervention, formal ethics committee review and informed consent were not required.

All data were extracted from openly available, official sources, such as TEG's website (https://www.enhanced.com/), World Athletics (https://worldathletics.org/athletes‐home), World Aquatics (https://www.worldaquatics.com/athletes and https://www.swimcloud.com/), Weightlifting sources (https://www.openweightlifting.org/search and https://iwf.sport/weightlifting_/athletes‐bios/) and official Olympic webpage (https://www.olympics.com/en/athletes/), all visited between May 24th and June 2nd of 2026. Specifically, whereas all TEG performances were derived from the official TEG website, individual historical performance data for all athletes were successfully gathered from the aforementioned websites, from which the following information was extracted: date of birth, nationality, sex, number of medals in international competitions (i.e., gold, silver and bronze), previous PB (i.e., absolute unit and date of occurrence). In addition, WR from each sports category and sex were extracted from those official websites, either current and previous WR. Of note, previous PB and WR performances were matched to TEG's sports facilities for track (i.e., straight 100 m lane), swimming (i.e., 50 m pool length) and weightlifting (i.e., body weight and modality categories).

TEG's sports categories varied according to each modality. Briefly, Track performances were restricted to 100 meters (100 m) divided between male and females. Swimming performances were restricted to the following: Freestyle (i.e., 50 m and 100 m for males and females), Breaststroke (i.e., 50 and 100 m for males), Fly (i.e., 50 m and 100 m for males), and Backstroke (i.e., 50 m for males). Weightlifting performances were composed of Snatch and Clean & Jerk modalities divided in different body weight categories for males (i.e., 110, 94, 88, and 79 kg) and females (i.e., 86 and 53 kg). In addition, there was a deadlift competition for two participants. Additionally, according to TEG's website information, all performance results were measured by internationally certified, approved and regulated equipment and officials, as any other Olympic event. These procedural details were reported by the organizers and could not be independently verified.

### 2.2 Data analysis

All performance analyses were carried out after full data extraction. The performances from Track and Swimming were expressed in seconds (s), whereas Weightlifting was expressed in kilograms (kg). Overall, performance parameters were analyzed as follows: (i) how many (and by how much) WR were broken; (ii) relative difference in performance (and difference in age) from each athlete's TEG and previous PB, which was further analyzed by grouping those new PB and non‐new PB performances, and according to PED exposure since some athletes self‐declared not taking any substance during TEG participation. Additionally, the relative difference between the two previous WR in each sport modality is also reported, in order to give proper context to the magnitude of performance improvement during TEG. Finally, we also investigated the association between the difference in age at PB and TEG (Δage in years) and difference in performance at PB and TEG (Δperformance in % change from PB) by means of a Linear Mixed‐Effects Model (LMM). The independent variable was defined as the Δage and the dependent variable was the Δperformance. To account for the dependency of repeated observations within the same athlete, athlete identity was entered into the model as a random intercept. Model parameters were estimated using Restricted Maximum Likelihood (REML) via the stats models library in Python. Statistical significance for the fixed effects (intercept and slope) was assessed at an alpha level of 0.05. Data are presented as mean ± standard deviation for continuous variables or as frequency and percentage for categorical variables, except otherwise stated. Whenever improvements in performance are considered, positive values reflect improved performance during TEG (when compared with either PB or WR). The 95% confidence intervals were calculated whenever there was subgroup analysis.

## 3 RESULTS

### 3.1 Overall sample characteristics

As shown in Table 1, a total of 42 athletes participated in the inaugural edition of TEG, generating 69 individual performance observations across three sporting modalities: Track, Swimming, and Weightlifting. Importantly, three weightlifting athletes (i.e., two females in Clean&Jerk and one male in Snatch categories) failed to achieve valid performances, thus 66 out of 69 performances were analyzed. Of the 42 competing athletes, four self‐declared as non‐enhanced (NE) at the time of competition. Overall, TEG's competitors were characterized by substantive international competitive experience, with a world class palmarès of gold, silver and bronze medals. After TEG's competition, 19 new PB were achieved across 12 enhanced athletes and 2 new PB were achieved from one NE athlete, all of whom competed in Swimming or Weightlifting; no Track athlete surpassed their historical PB. A single WR was broken during the event, recorded by an enhanced male swimmer in the 50 m freestyle event. The sample characteristics of only enhanced and NE athletes are shown in Tables S1 and S2, respectively.

**TABLE 1:** Overall sample characteristics of athletes competing in TEG.

	Swimming	Track	Weightlifting
Total athletes [*n*, %males]	18 (77.8%)	13 (53.8%)	11 (72.7%)
Total Enhanced Athletes [*n* (%)]	16 (88.9%)	11 (84.6%)	11 (100.0%)
Age (years)	31.67 ± 4.68	30.92 ± 2.66	32.73 ± 3.80
Palmar*è*s [median (min–max)]			
Gold medals	1 (0–17)	2 (0–25)	7 (0–62)
Silver medals	0 (0–22)	0 (0–4)	2 (0–15)
Bronze medals	2 (0–21)	1 (0–3)	2 (0–11)
Athletes with TEG's new PB	10	0	3
Total of TEG's new PB	17*	0	4
Total of WR	1**	0	0

*Note*: Palmarès includes medals from international competitions and is expressed as median (minimum–maximum).

Abbreviations: PB, personal best performance; SD, standard deviation; TEG, The Enhanced Games; WR, world record.

* Includes two PB achieved by a self‐declared non‐enhanced athlete (50 m Backstroke and 100 m Butterfly).

** World record set by a male swimmer in the 50 m freestyle event using a prohibited in World Aquatics official competitions polyurethane‐swimming‐suit.

### 3.2 World record performance achievements

As shown in Table 2, across all performance observations from enhanced athletes, pre‐TEG PB were approximately 6% below the current WR at the time of competition. After the event, TEG's performances were systematically more distant from their respective sports modality WR, indicating, on average, worse performances than their own historical PB This deterioration was most pronounced in Track, followed by Weightlifting and Swimming performances. Swimming was the sole modality in which the overall TEG‐to‐WR gap remained comparable to the historical PB‐to‐WR gap. The same trend was observed when the sample was filtered for either NE or combining both athletes' categories, as shown in Tables S3 and S4.

**TABLE 2:** Enhanced athletes' PB and TEG performance in comparison to current WR.

Subgroup	PB to WR difference (%, average ± SD [95% CI])	*n*	TEG to WR difference (%, average ± SD [95% CI])	*n*
Overall	−5.92% ± 4.68% [−7.10% to −4.74%]	63	−8.91% ± 7.52% [−10.85% to −6.96%]	60
Females	−7.10% ± 3.36% [−8.72% to −5.48%]	19	−10.26% ± 6.31% [−13.50% to −7.01%]	17
Males	−5.41% ± 5.09% [−6.96% to −3.86%]	44	−8.37% ± 7.95% [−10.82% to −5.92%]	43
Track	−5.12% ± 1.61% [−6.20% to −4.04%]	11	−11.37% ± 5.80% [−15.27% to −7.48%]	11
Females	−6.04% ± 1.49% [−7.90% to −4.19%]	5	−13.52% ± 7.50% [−22.83% to −4.21%]	5
Males	−4.35% ± 1.34% [−5.76% to −2.94%]	6	−9.59% ± 3.72% [−13.49% to −5.68%]	6
Swimming	−4.59% ± 3.86% [−5.98% to −3.20%]	32	−5.11% ± 3.13% [−6.24% to −3.98%]	32
Females	−6.98% ± 2.26% [−8.87% to −5.08%]	8	−6.69% ± 3.38% [−9.51% to −3.86%]	8
Males	−3.80% ± 3.98% [−5.48% to −2.11%]	24	−4.59% ± 2.93% [−5.82% to −3.35%]	24
Weightlifting	−8.48% ± 5.96% [−11.27% to −5.69%]	20	−14.45% ± 10.14% [−19.67% to −9.24%]	17
Females	−8.16% ± 5.40% [−13.83% to −2.48%]	6	−13.32% ± 6.80% [−24.13% to −2.50%]	4
Males	−8.62% ± 6.38% [−12.30% to −4.94%]	14	−14.80% ± 11.18% [−21.56% to −8.05%]	13

*Note*: Values represent the mean percentage difference (and standard deviation, SD) relative to the current WR, accompanied by 95% confidence intervals (CI). Positive values indicate performance above the current WR.

Abbreviations: PB, personal best; TEG, The Enhanced Games; WR, world record.

The current WR performances utilized for the abovementioned calculations are shown in the Supplementary Material Table S5. Based on these values, the average difference between current and immediately previous WR is 0.93 ± 0.77% (i.e., Females, 1.17 ± 1.24%; Males, 0.85 ± 0.63%). Specifically, differences between current and previous WR for Track and Swimming are 1.86 ± 1.01% (Females, 2.57%; Males, 1.15%), 0.63 ± 0.52% (Females, 0.47 ± 0.30%; Males, 0.67 ± 0.58%), respectively. Importantly, this comparison was not possible for Weightlifting, as the current sports categories were recently changed. Nonetheless, for deadlift competition, the difference between current and previous WR is 1.76%.

### 3.3 Personal best performance achievements

As shown in Table 3, across all 60 enhanced athletes' performance observations, TEG performances were on average worse than each athlete's pre‐existing historical PB. Whereas 19 new PBs were achieved, the remaining 41 observations reflected a mean decline in comparison to previous PB.

**TABLE 3:** Enhanced TEG athletes' performances differences between TEG and previous PB, stratified by modality, sex, and PB outcome.

Subgroup	Overall	*n*	New PB at TEG	*n*	Non‐new PB at TEG	*n*
Overall	−2.86% ± 7.39% [−4.77% to −0.95%]	60	3.09% ± 2.59% [1.84% to 4.34%]	19	−5.61% ± 7.28% [−7.91% to −3.31%]	41
Female	−2.95% ± 6.41% [−6.25% to 0.34%]	17	4.26% ± 2.34% [0.54% to 7.99%]	4	−5.18% ± 5.54% [−8.52% to −1.83%]	13
Male	−2.82% ± 7.82% [−5.22% to −0.41%]	43	2.78% ± 2.64% [1.32% to 4.24%]	15	−5.81% ± 8.05% [−8.94% to −2.69%]	28
Track	−5.94% ± 5.16% [−9.41% to −2.48%]	11	—	0	−5.94% ± 5.16% [−9.41% to −2.48%]	11
Female	−7.07% ± 7.34% [−16.18% to 2.04%]	5	—	0	−7.07% ± 7.34% [−16.18% to 2.04%]	5
Male	−5.01% ± 2.79% [−7.93% to −2.08%]	6	—	0	−5.01% ± 2.79% [−7.93% to −2.08%]	6
Swimming	−0.20% ± 2.98% [−1.28% to 0.87%]	32	2.25% ± 2.12% [1.08% to 3.42%]	15	−2.37% ± 1.61% [−3.20% to −1.54%]	17
Female	0.26% ± 2.90% [−2.17% to 2.68%]	8	3.26% ± 1.47% [−0.40% to 6.92%]	3	−1.54% ± 1.68% [−3.63% to 0.54%]	5
Male	−0.36% ± 3.05% [−1.64% to 0.93%]	24	2.00% ± 2.23% [0.58% to 3.41%]	12	−2.71% ± 1.53% [−3.68% to −1.74%]	12
Weightlifting	−5.85% ± 11.73% [−11.88% to 0.18%]	17	6.24% ± 1.60% [3.69% to 8.79%]	4	−9.57% ± 10.92% [−16.17% to −2.97%]	13
Female	−4.23% ± 8.35% [−17.52% to 9.05%]	4	7.27% ± — [—]	1	−8.07% ± 4.03% [−18.08% to 1.94%]	3
Male	−6.35% ± 12.85% [−14.11% to 1.41%]	13	5.89% ± 1.77% [1.49% to 10.30%]	3	−10.02% ± 12.43% [−18.91% to −1.13%]	10

*Note*: Values represent the mean percentage difference (and standard deviation, SD) relative to previous PB performance, accompanied by 95% confidence intervals (CI). Positive values indicate performance above previous PB.

Abbreviations: PB, personal best; TEG, The Enhanced Games.

Based on Table 3 results (i.e., only enhanced athletes considered), at the modality level, Track was characterized by a uniform failure to improve upon previous PB, thus resulting in none new PB. On the other hand, Swimming yielded the most heterogeneous improved results overall, with female swimmers demonstrating a marginal aggregate improvement. Finally, Weightlifting athletes exhibited the greatest inter‐individual variability, as three athletes achieved new PB, while the remaining 8 showed decreased performances relative to their previous PB. Individual differences between PB and TEG's performances can be seen in Supplementary Material for Track (i.e., Figure S1), Swimming (i.e., Figures S2 and S3) and Weightlifting (Figure S3, panel D). The same trend was found when filtering by NE or combining both athletes' categories, as shown in Tables S6 and S7, respectively.

Table 4 shows that those enhanced athletes with new PB at TEG were younger than those without a new PB at TEG, a difference consistent with, but not demonstrative of, an age‐related decline in performance despite PED use. Again, the same trend was found when filtering by NE or combining both athletes' categories, as shown in Tables S8 and S9, respectively.

**TABLE 4:** Enhanced TEG athletes' age at previous PB and difference in age between PB and TEG (Δage), stratified by PB achievement status (new PB vs. no new PB).

Subgroup	New PB performances		*n*	Non‐new PB performances		*n*
	Age at PB (years)	Δage (years)		Age at PB (years)	Δage (years)	
Overall	25.42 ± 4.17 [23.41–27.43]	5.37 ± 3.30 [3.78–6.96]	19	26.02 ± 4.32 [24.71–27.34]	7.09 ± 3.81 [5.93–8.25]	44
Track	—	—	0	26.27 ± 3.41 [23.98 to 28.56]	4.91 ± 3.14 [2.80 to 7.02]	11
Swimming	24.60 ± 4.00 [22.39–26.81]	6.07 ± 3.35 [4.21–7.92]	15	26.59 ± 4.69 [24.18–29.00]	7.88 ± 3.90 [5.88–9.89]	17
Weightlifting	28.50 ± 3.70 [22.62–34.38]	2.75 ± 1.26 [0.75–4.75]	4	25.25 ± 4.61 [22.79–27.71]	7.75 ± 3.77 [5.74–9.76]	16

*Note*: Δage, difference in years between age at TEG competition and age at which the previous PB was achieved.

Abbreviations: PB, personal best; TEG, The Enhanced Games.

### 3.4 Age‐related performance changes

As shown in Figure 1, TEG enhancing protocol was not associated with an attenuation of the expected age‐related decline in athletic performance; the slope of the relationship was negative but did not reach statistical significance (*p* = 0.26). The summary data utilized to build the current analysis are shown in Tables 3 and 5 for Δperformance and Δage, respectively. The same analysis was performed by filtering the data for NE (i.e., Tables S8 and S10; Figure S4) or combining both athletes categories (i.e., Tables S9 and S11; Figure S5), which showed the same trend as the enhanced athletes' outcomes.

**FIGURE 1:**
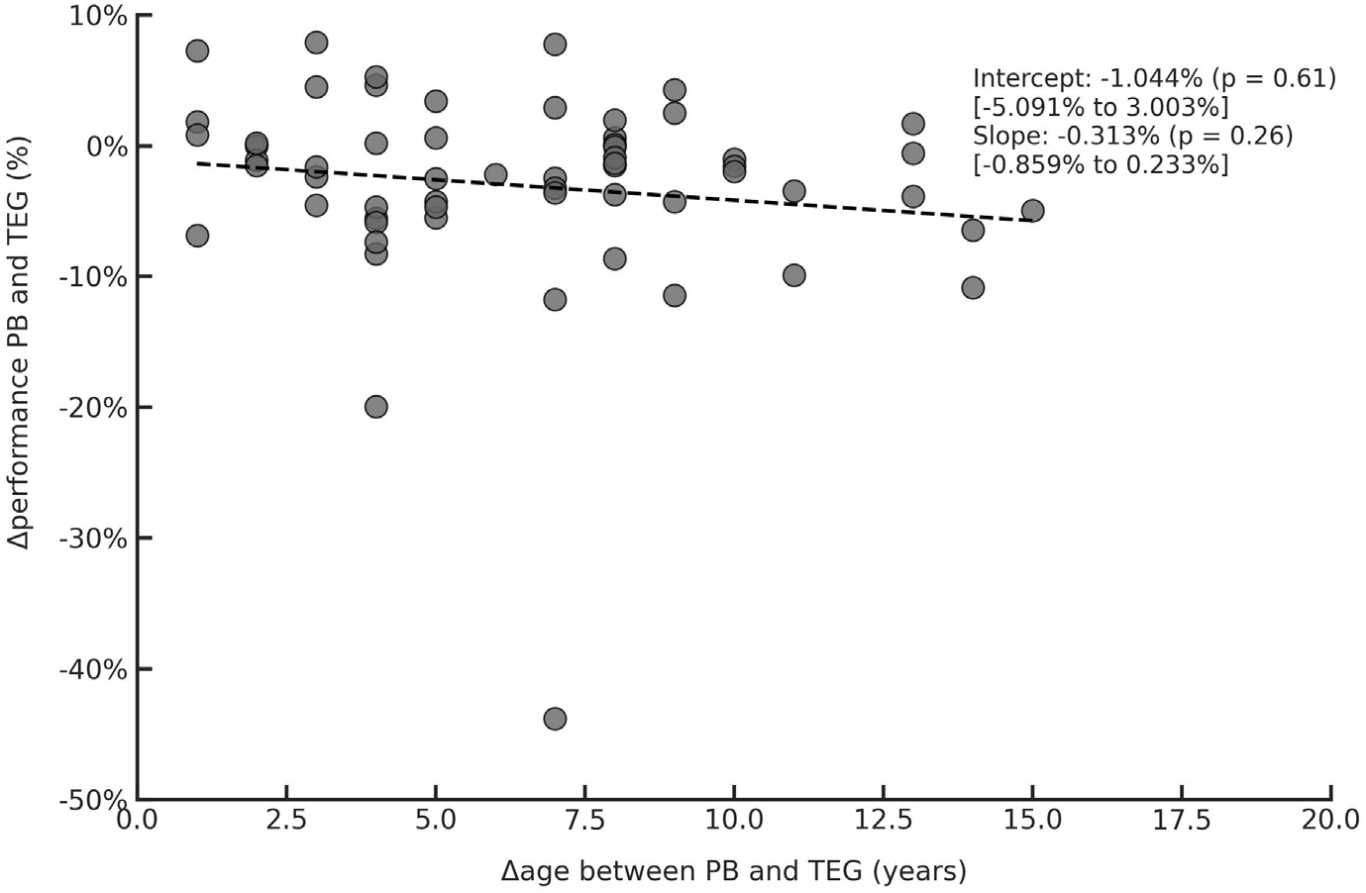
Correlation between the age and performance differences before and after TEG. TEG, The Enhanced Games; *y*‐axis expresses the relative difference between previous PB and TEG performance (positive values indicate increased performance at TEG when compared to PB); *x*‐axis expresses the age difference (in years) between age at TEG and age at previous PB. Slope and intercept accompanied by *p*‐values (between parenthesis) and 95% confidence intervals (between brackets) are presented.

**TABLE 5:** Enhanced TEG athletes' age at PB and age difference between PB and TEG's performances, stratified by modality and sex.

Subgroup	Age at PB (years)	Δage (years)
Overall (*n* = 63)	25.84 ± 4.25 [24.77–26.91]	6.57 ± 3.73 [5.63–7.51]
Females (*n* = 19)	24.95 ± 2.66 [23.67–26.23]	7.47 ± 4.38 [5.36–9.58]
Males (*n* = 44)	26.23 ± 4.75 [24.78–27.67]	6.18 ± 3.39 [5.15–7.21]
Track (*n* = 11)	26.27 ± 3.41 [23.98–28.56]	4.91 ± 3.14 [2.80–7.02]
Females (*n* = 5)	26.00 ± 2.12 [23.37–28.63]	4.00 ± 0.71 [3.12–4.88]
Males (*n* = 6)	26.50 ± 4.42 [21.87–31.13]	5.67 ± 4.23 [1.23–10.10]
Swimming (*n* = 32)	25.66 ± 4.43 [24.06–27.25]	7.03 ± 3.71 [5.69–8.37]
Females (*n* = 8)	24.00 ± 1.41 [22.82–25.18]	8.75 ± 3.28 [6.00–11.50]
Males (*n* = 24)	26.21 ± 4.95 [24.12–28.30]	6.46 ± 3.73 [4.88–8.03]
Weightlifting (*n* = 20)	25.90 ± 4.55 [23.77–28.03]	6.75 ± 3.96 [4.90–8.60]
Females (*n* = 6)	25.33 ± 4.03 [21.10–29.57]	8.67 ± 6.09 [2.28–15.06]
Males (*n* = 14)	26.14 ± 4.88 [23.32–28.96]	5.93 ± 2.50 [4.49–7.37]

*Note*: Δage, difference in years between age at TEG competition and age at which the previous PB was achieved.

Abbreviations: PB, personal best; TEG, The Enhanced Games.

## 4 DISCUSSION

Based on the current results, it could be postulated that TEG failed to deliver on their three core promises, which were (i) generating new WR; (ii) enabling athletes to exceed their previous PB; and (iii) attenuating the expected age‐related declines in athletic performance. Whereas the so‐called enhanced athletes were not able to break a significant amount of WR, some new PB in Swimming and Weightlifting sport modalities were achieved; nonetheless, some of these results were from a non‐enhanced athlete. Finally, even with the new WR and some PB marks, the overall athletic outcomes were worse than PB marks, thus suggesting that TEG's protocols were not able to counteract a possible negative aging effect and/or lack of high‐quality training regimen. Taken together, the current analysis shows, for the first time, that TEG's performances were not at the level expected from TEG's public performance claims, despite a considerable amount of new PB from past‐prime physical condition in some athletes.

### 4.1 TEG's world record performance achievements

As overtly advertised from TEG's media, the breaking of WR was fundamental for its success as a business. However, based on the current data, TEG's performances resulted in substantially fewer WR achievements than anticipated from the event's public claims. It could be speculated that this “failure” from TEG's claims perspective might be related to the pool of athletes entering the competition, rather than the PED protocols adopted.

TEG athletes, as shown in Table 1, could be described as world‐class. However, based on their previous individual PB, these athletes were ~6% worse than the current WR, which is a considerably far greater gap usually observed between two sequential WR (which is usually less than 1%). Interestingly, after TEG, the gap to WR records actually increased to ~9% regardless of sex or PED exposure, though it varied according to the sports modality. Of note, whereas Track and Weightlifting performances got worse in comparison to WR after TEG, Swimming athletes seemed to have benefited. Importantly, it is uncertain if PED protocols were actually effective only in Swimming as the so‐called “banned‐suits” were allowed during TEG, even during the sole WR breaking (i.e., 50 m freestyle male performance). Of note, the WR in 50‐m freestyle for males was recently broken (i.e., March 20th, 2026) under the World Anti‐Doping Agency (WADA)'s and World Aquatics' regulations, thus without the “banned‐suit,” which suggests that promised gains with PED protocols were not as effective as expected.

According to TEG's clinical trial available data (i.e., registration in clinical trials website #ASCEND001), PED protocol consisted on 25 weeks of several AAS substances (e.g., Testosterone Enanthate, Cypionate, and Propionate, as well as other testosterone derivatives) and other PED such as growth hormone and erythropoietin. Overall, it was reported that almost all athletes involved in the trial used testosterone or testosterone esters, more than half utilized stimulants, and a minority used erythropoietin and other AAS (Boardley, 2026). Indeed, these substances are known to promote gains in physical performance (Watson et al., 2022) and compose the prohibited WADA list (Petróczi & Blank, 2025). However, no information is available on the actual doses and exact substances utilized in TEG's PED protocol, which could impact the interpretation of current results. For example, it is unknown whether AAS were administered only within clinically recommended doses, in which case might have not been enough for performance enhancement (Bhasin & Snyder, 2025). In addition, the undeniable gains in muscle mass and force from anabolic steroids might not always transfer for athletic performance (Andrews et al., 2018; Bhasin et al., 1996).

### 4.2 PB performance achievements

From an individual performance perspective, perhaps the most notable outcome from TEG's results was the 19 new PB (out of 60 performances) from 12 enhanced athletes (i.e., see Table 2 for details). Indeed, this achievement could be considered remarkable, as the majority of TEG's athletes were at past‐athletic prime period of their careers. Thus, the PED protocol might have positively influenced some but not all TEG's performances.

When TEG's performances were divided into new vs non‐new PB performances, the former group improved by ~3% relative to the previous PB, whereas the latter presented an ~5% worsened performance. Importantly, this discrepancy between groups might have been skewed by Track outcomes, as there were no new PB achievements in Track modalities. The reasons for the lack of new PB in Track are uncertain. A possible explanation could be that the pool of Track athletes were even further away from their prime physical level compared to Swimming and Weightlifting samples. Additionally, it could be that the PED protocols and/or sports‐related enhancing technologies utilized in Track were not as effective as in Swimming, which deliberately utilized banned suits. Potentially, these discrepancies across sports modalities may indicate that PED protocols are highly specific to the dynamics of the events (e.g., hydro‐dynamic vs. aerodynamics).

At least partially, the new PB might have been driven by the age of these athletes in combination with TEG's protocol. For example, when comparing new and non‐new PB groups, the average age at TEG was somewhat closer to PB's age in the former group (~5 vs. ~7 years in the non‐new PB group). Although the sample size is considerably small, there is a nuance between the different sports. Whereas those with new PB showed a gap difference of only ~3 years (vs. ~8 years in non‐new PB) in Weightlifting, Swimming athletes with and without new PB showed a much lower gap in age.

Of note, the peak performance at these modalities generally occurs at between early to mid‐20's (Agudo‐Ortega et al., 2024; Šiljeg et al., 2024; Solberg et al., 2019). Therefore, a smaller age gap between TEG and PB might have played a larger role in the new performance marks rather than the sole enhancement performance effect from the drugs utilized. For example, Track, Weightlifting or Swimming peak performance occurs around 25 years of age for both sexes (Agudo‐Ortega et al., 2024; Šiljeg et al., 2024; Solberg et al., 2019; Tanaka & Seals, 1997); thus, if TEG's protocol were to be extremely effective, the current athlete's performances should, theoretically, be better. Notably, this was not the case for the majority of the athletes. Another potential explanation for the differences in performances between new and non‐new PB groups could be a potential interference from a lack of continuous high‐quality training periods in the latter group. Briefly, athletic performance decrease might also be influenced by poorer preparation because of repeated injuries and/or training gaps related to other personal reasons rather than the negative aging‐related effects in physical capacities. Because of the lack of data available from TEG's athletes' preparation for the event participation, any conclusion regarding the difference between these two groups remains speculative.

### 4.3 Age‐related performance outcomes at TEG

In line with the previous discussion, it is known that the majority of physical capacities decline after the third decade of life, such as maximal strength, power and aerobic capacities (Gries et al., 2018; Hunter et al., 2016). A relevant claim from TEG's protocol with PED is to counteract the negative aging effects in physical performance (Cox & Khan, 2025; Sandbakk & Loland, 2026). Herein, we examined whether the relationship between age and performance outcomes observed at TEG was consistent with this claim. No evidence was found for improved performance with increasing Δage. Instead, the overall trend actually suggested poorer performance outcomes as the time elapsed since athletes' previous PB increased. It is noteworthy that the association between Δage and Δperformance did not reach statistical significance (i.e., *p* = 0.26), which may only reinforce the potential lack of anti‐aging effect from PED utilization in athletic performance. It is worth mentioning that some TEG's athletes potentially benefited from PED effects despite years off their athletic prime results, as shown in individual data points in Figure 1. Therefore, caution is needed when interpreting these results, since factors other than sole aging effect (i.e., as highlighted in the previous topic) could directly impact TEG's performances and the current study did not compare the outcomes with an age‐matched controlled group. Nonetheless, it could be speculated, based on the direction of the observed relationship, the overall results suggest an inconsistency with TEG's expectation that PED use would offset a potential age‐related decline in athletic performance. Therefore, when the overall data is analyzed, there is no evidence supporting the claim that TEG's enhancing protocols could substantially attenuate the decline in performance typically observed with aging or compensate for a lack of high‐quality training regimens.

### 4.4 Performance outcomes among non‐enhanced athletes

Interestingly, 6 out of the 69 performances analyzed were achieved by athletes who reportedly did not undergo TEG's PED protocols. Despite their small number, most of these NE performances were actually closer to athletes' PB (~ −1%) when compared to “enhanced” athletes' performance gap (~ −3%). For instance, a NE swimmer achieved two new PB, whereas among sprinters, a NE athlete produced the performance closest to a new PB (Figures S1 and S2). Although these observations should be interpreted cautiously, they reinforce the notion that elite athletic performance is multifactorial and not solely determined by pharmacological enhancement.

## 5 LIMITATIONS

Some limitations should be acknowledged. First, PED exposure was not independently verified, and information regarding dose, duration, and adherence was unavailable. In addition, it is uncertain, based on publicly available data, which specific “performance‐enhancing technologies” were utilized by each athlete (as discussed in *TEG's world record performance achievements*). Second, the retrospective and observational nature of the study precludes causal inference regarding the efficacy of specific PED protocols. Thus, caution is needed when interpreting these unverified secondary data, especially among self‐declared NE athletes. For example, self‐declared “non‐enhanced” athletes might actually be using substances, or enhanced athletes might be using highly sub‐optimal dosages, which artificially deflates the apparent effectiveness of the protocols. Third, the absence of an age‐matched control group without PED exposure makes it difficult to definitively isolate the effects of TEG's protocols from natural age‐related decline (as discussed in *Age‐related performance outcomes at TEG*). Finally, most athletes evaluated were past their physical prime; consequently, the observed performance gaps may be confounded by a lack of continuous, high‐quality training regimens rather than pharmacological efficacy alone (as discussed in *PB performance achievements*).

## 6 CONCLUSION

In summary, TEG demonstrated that the implementation of systematic PED protocols and removal of anti‐doping regulations did not consistently translate into superior athletic performance, as initially suggested by the organizers. Thus, by revisiting the organization's core premises, our data indicate that TEG failed to (i) generate new WR; (ii) enable athletes to exceed their previous PB; and (iii) attenuate the expected age‐related declines in athletic performance. Despite bold assertions of extending human physical capabilities and new WR, only a single record was broken, and the overall performance largely deteriorated across sports modalities when compared to athletes' own historical PB. Of note, it is worth mentioning that some TEG's athletes could indeed achieve new PB, even past their athletic prime period. Nonetheless, the collective data provide no evidence that these protocols attenuated age‐related and/or lack of high‐quality training periods that might negatively impact athletic performance. Taken together, these findings challenge key performance‐related claims underlying TEG model and suggest that PED use alone is insufficient to reliably overcome the multifactorial determinants of elite athletic performance.

## AUTHOR CONTRIBUTIONS

**Rafael A. Azevedo:** Conceptualization; data curation; formal analysis; funding acquisition; investigation; methodology; project administration; resources; software; supervision; validation; visualization. **Luis F. Coutinho:** Conceptualization; data curation; formal analysis; funding acquisition; investigation; methodology; project administration; resources; software; supervision; validation; visualization. **Monique C. Gonçalves:** Conceptualization; data curation; formal analysis; funding acquisition; investigation; methodology; project administration; resources; software; supervision; validation; visualization. **Higor A. Oliveira:** Conceptualization; data curation; formal analysis; funding acquisition; investigation; methodology; project administration; resources; software; supervision; validation; visualization. **Guilherme J. Gonçalves:** Conceptualization; data curation; formal analysis; funding acquisition; investigation; methodology; project administration; resources; software; supervision; validation; visualization. **Marcus Campos:** Validation; visualization. **Hamilton Roschel:** Validation; visualization. **Bruno Gualano:** Conceptualization; validation; visualization.

## FUNDING INFORMATION

This study was funded, in part, by scholarships provided by: Coordenação de Aperfeiçoamento de Pessoal de Nível Superior ‐ Brazil (CAPES) ‐ Finance Code 001 to Monique C. Gonçalves; and São Paulo Research Foundation to Rafael A. Azevedo (FAPESP; grant number 2023/10778‐0).

## CONFLICT OF INTEREST STATEMENT

The authors declare no conflicts of interest.

## ETHICS STATEMENT

This study is a retrospective analysis utilizing publicly available, secondary data obtained from official websites and open registries. In accordance with institutional and national guidelines for observational research relying exclusively on public domain data without direct human contact or intervention, formal ethics committee review and informed consent were not required/waived.

## Supporting information


**Table S1.** Enhanced athletes' characteristics competing in TEG.
**Table S2.** Non‐enhanced athletes' characteristics competing in TEG.
**Table S3.** Non‐enhanced TEG athletes' PB and TEG performance in comparison to current WR.
**Table S4.** Overall TEG athletes' PB and TEG performance in comparison to current WR.
**Table S5.** Current and previous World Records from each sports modality, divided by sex and categories.
**Table S6.** Non‐enhanced TEG athletes' performances differences between TEG and previous PB, stratified by modality, sex, and PB outcome.
**Table S7.** Overall TEG athletes' performances differences between TEG and previous PB, stratified by modality, sex, and PB outcome.
**Table S8.** Non‐enhanced TEG athletes' age at previous PB and difference in age between PB and TEG (Δage), stratified by PB achievement status (new PB vs. no new PB).
**Table S9.** Overall TEG athletes' age at previous PB and difference in age between PB and TEG (Δage), stratified by PB achievement status (new PB vs. no new PB).
**Table S10.** Non‐enhanced TEG athletes' age at PB and age difference between PB and TEG's performances, stratified by modality and sex.
**Table S11.** Overall TEG athletes' age at PB and age difference between PB and TEG's performances, stratified by modality and sex.
**Figure S1.** 100 m track sprint performance for females and males.
**Figure S2.** 50 and 100 m Swimming freestyle performance for females and males.
**Figure S3.** Swimming and Weightlifting performances for females and males.
**Figure S4.** Correlation between the age and performance differences before and after TEG for non‐enhanced athletes.
**Figure S5.** Correlation between the age and performance differences before and after TEG for all athletes.

## Data Availability

The datasets analyzed during the current study are available in publicly accessible repositories and official websites, including The Enhanced Games website (https://www.enhanced.com/), World Athletics (https://worldathletics.org/), World Aquatics (https://www.worldaquatics.com/), SwimCloud (https://www.swimcloud.com/), OpenWeightlifting (https://www.openweightlifting.org/), the International Weightlifting Federation (https://iwf.sport/), and the official Olympic website (https://www.olympics.com/). Derived data supporting the findings of this study are available from the corresponding author upon reasonable request.
