# Supplementary material for: Enhanced but not superior: Performance outcomes from the inaugural enhanced games
Source: Physiol Rep. 2026 Sep 29;14(19):e71123. doi: 10.14814/phy2.71123 (PMC13623661; doi:10.14814/phy2.71123)
Supplement: Supplementary file 1 [file PHY2-14-e71123-s001.docx]

**Supplementary material**

**Title:** Enhanced but not superior: performance outcomes from the inaugural Enhanced Games

**Authors:** Rafael A. Azevedo^1,2^, Luis F. Coutinho^3^, Monique C. Gonçalves^1,2^, Higor A. Oliveira^1,2^, Guilherme J. Gonçalves^1,2^, Hamilton Roschel^1,2^, Bruno Gualano^1,2^

^1^Applied Physiology and Nutrition Research Group, School of Physical Education and Sport, Faculdade de Medicina FMUSP, Universidade de São Paulo, São Paulo, Brazil.

^2^Center of Lifestyle Medicine, Faculdade de Medicina FMUSP, Universidade de São Paulo, São Paulo, Brazil.

^3^Faculty of Medical Sciences of Minas Gerais (FC MMG), Belo Horizonte, Brazil

**Correspondence:** gualano@usp.br

Table of contents

[Table S1. Enhanced athletes’ characteristics competing in TEG.	2](#_Toc239251089)

[Table S2. Non-enhanced athletes’ characteristics competing in TEG.	2](#_Toc239251090)

[Table S3. Non-enhanced TEG athletes’ PB and TEG performance in comparison to current WR.	3](#_Toc239251091)

[Table S4. Overall TEG athletes’ PB and TEG performance in comparison to current WR.	4](#_Toc239251092)

[Table S5. Current and previous World Records from each sports modality, divided by sex and categories	5](#_Toc239251093)

[Table S6. Non-enhanced TEG athletes’ performances differences between TEG and previous PB, stratified by modality, sex, and PB outcome.	7](#_Toc239251094)

[Table S7. Overall TEG athletes’ performances differences between TEG and previous PB, stratified by modality, sex, and PB outcome.	8](#_Toc239251095)

[Table S8 – Non-enhanced TEG athletes’ age at previous PB and difference in age between PB and TEG (Δage), stratified by PB achievement status (new PB vs. no new PB).	9](#_Toc239251096)

[Table S9 – Overall TEG athletes’ age at previous PB and difference in age between PB and TEG (Δage), stratified by PB achievement status (new PB vs. no new PB).	10](#_Toc239251097)

[Table S10. Non-enhanced TEG athletes’ age at PB and age difference between PB and TEG’s performances, stratified by modality and sex.	11](#_Toc239251098)

[Table S11. Overall TEG athletes’ age at PB and age difference between PB and TEG’s performances, stratified by modality and sex.	12](#_Toc239251099)

[Figure S1. 100m track sprint performance for females and males.	13](#_Toc239251100)

[Figure S2. 50 and 100m Swimming freestyle performance for females and males.	14](#_Toc239251101)

[Figure S3. Swimming and Weightlifting performances for females and males.	15](#_Toc239251102)

[Figure S4. Correlation between the age and performance differences before and after TEG for non-enhanced athletes.	16](#_Toc239251103)

[Figure S5. Correlation between the age and performance differences before and after TEG for all athletes.	16](#_Toc239251104)

# Table S1. Enhanced athletes’ characteristics competing in TEG.

|  | **Swimming** | **Track** | **Weightlifting** |
| --- | --- | --- | --- |
| Total athletes [n, %males] | 16 (75.0%) | 11 (54.5%) | 11 (72.7%) |
| Total Enhanced Athletes [n (%)] | 16 (100.0%) | 11 (100.0%) | 11 (100.0%) |
| Age (years) | 32.69 ± 3.81 | 31.18 ± 2.75 | 32.73 ± 3.80 |
| Palmar*è*s [median (min–max)] |  |  |  |
| Gold medals | 1 (0 - 17) | 2 (0 - 9) | 7 (0 - 62) |
| Silver medals | 0 (0 - 22) | 0 (0 - 3) | 2 (0 - 15) |
| Bronze medals | 2 (0 - 21) | 1 (0 - 3) | 2 (0 - 11) |
| Athletes with TEG’s new PB | 9 | 0 | 3 |
| Total of TEG’s new PB | 15 | 0 | 4 |
| Total of WR | 1 | 0 | 0 |

*TEG, The Enhanced Games; PB, personal best performance; WR, world record; SD, standard deviation. Palmarès includes medals from international competitions and is expressed as median (minimum–maximum). **World record set by a male swimmer in the 50m freestyle event using a prohibited in World Aquatics official competitions polyurethane-swimming-suit.*

# Table S2. Non-enhanced athletes’ characteristics competing in TEG.

|  | **Swimming** | **Track** | **Weightlifting** |
| --- | --- | --- | --- |
| Total athletes [n, %males] | 2 (100.0%) | 2 (50.0%) | — |
| Total Enhanced Athletes [n (%)] | — | — | — |
| Age (years) | 23.50 ± 2.12 | 29.50 ± 2.12 | — |
| Palmar*è*s [median (min–max)] |  |  |  |
| Gold medals | 5 (0 - 10) | 13 (1 - 25) | — |
| Silver medals | 2 (0 - 5) | 2 (0 - 4) | — |
| Bronze medals | 2 (0 - 5) | 1 (0 - 2) | — |
| Athletes with TEG’s new PB | 1 | 0 | — |
| Total of TEG’s new PB | 2 | 0 | — |
| Total of WR | 0 | 0 | — |

*TEG, The Enhanced Games; PB, personal best performance; WR, world record; SD, standard deviation. Palmarès includes medals from international competitions and is expressed as median (minimum–maximum). *PB achieved by a self-declared non-enhanced athlete (50m Backstroke and 100m Butterfly).*

# Table S3. Non-enhanced TEG athletes’ PB and TEG performance in comparison to current WR.

| **Subgroup** | **PB to WR difference**  **(%, average ± SD [95% CI])** | **n** |  | **TEG to WR difference**  **(%, average ± SD [95% CI])** | **n** |
| --- | --- | --- | --- | --- | --- |
| **Overall** | -4.73% ± 3.65%  [-8.57% to -0.90%] | 6 |  | -5.42% ± 2.38%  [-7.93% to -2.92%] | 6 |
| Females | -6.20% ± —  [—] | 1 |  | -7.24% ± —  [—] | 1 |
| Males | -4.44% ± 4.00%  [-9.41% to 0.53%] | 5 |  | -5.06% ± 2.47%  [-8.13% to -1.99%] | 5 |
| **Track** | -4.04% ± 3.05%  [-31.47% to 23.39%] | 2 |  | -5.66% ± 2.24%  [-25.82% to 14.51%] | 2 |
| Females | -6.20% ± —  [—] | 1 |  | -7.24% ± —  [—] | 1 |
| Males | -1.88% ± —  [—] | 1 |  | -4.07% ± —  [—] | 1 |
| **Swimming** | -5.08% ± 4.32%  [-11.95% to 1.79%] | 4 |  | -5.31% ± 2.78%  [-9.73% to -0.88%] | 4 |
| Females | — | 0 |  | — | 0 |
| Males | -5.08% ± 4.32%  [-11.95% to 1.79%] | 4 |  | -5.31% ± 2.78%  [-9.73% to -0.88%] | 4 |
| **Weightlifting** | — | 0 |  | — | 0 |
| Females | — | 0 |  | — | 0 |
| Males | — | 0 |  | — | 0 |

*PB, personal best; TEG, The Enhanced Games; WR, world record; Values represent the mean percentage difference (and standard deviation, SD) relative to the current WR, accompanied by 95% confidence intervals (CI). Positive values indicate performance above the current WR.*

# Table S4. Overall TEG athletes’ PB and TEG performance in comparison to current WR.

| **Subgroup** | **PB to WR difference**  **(%, average ± SD [95% CI])** | **n** |  | **TEG to WR difference**  **(%, average ± SD [95% CI])** | **n** |
| --- | --- | --- | --- | --- | --- |
| **Overall** | -5.82% ± 4.59%  [-6.92%, -4.70%] | 69 |  | -8.59% ± 7.27%  [-10.38% to -6.80%] | 66 |
| Females | -7.06% ± 3.28%  [-8.59% to -5.52%] | 20 |  | -10.09% ± 6.16%  [-13.15% to -7.03%] | 18 |
| Males | -5.31% ± 4.97%  [-6.73%, -3.88%] | 49 |  | -8.03% ± 7.62%  [-10.24% to -5.81%] | 48 |
| **Track** | -4.95% ± 1.76%  [-6.02% to -3.89%] | 13 |  | -10.49% ± 5.75%  [-13.97% to -7.02%] | 13 |
| Females | -6.07% ± 1.34%  [-7.47% to -4.67%] | 6 |  | -12.47% ± 7.18%  [-20.00% to -4.94%] | 6 |
| Males | -4.00% ± 1.54%  [-5.42% to -2.57%] | 7 |  | -8.80% ± 3.99%  [-12.48% to -5.11%] | 7 |
| **Swimming** | -4.64% ± 3.85%  [-5.95%, -3.33%] | 36 |  | -5.13% ± 3.06%  [-6.17% to -4.10%] | 36 |
| Females | -6.98% ± 2.26%  [-8.87% to -5.08%] | 8 |  | -6.69% ± 3.38%  [-9.51% to -3.86%] | 8 |
| Males | -3.98% ± 3.97%  [-5.52%, -2.43%] | 28 |  | -4.69% ± 2.87%  [-5.80% to 3.58%] | 28 |
| **Weightlifting** | -8.48% ± 5.96%  [-11.27% to 5.69%] | 20 |  | -14.45% ± 10.14%  [-19.67% to 9.24%] | 17 |
| Females | -8.16% ± 5.40%  [-13.83% to -2.48%] | 6 |  | -13.32% ± 6.80%  [-24.13% to -2.50%] | 4 |
| Males | -8.62% ± 6.38%  [-12.30% to -4.94%] | 14 |  | -14.80% ± 11.18%  [-21.56% to -8.05%] | 13 |

*PB, personal best; TEG, The Enhanced Games; WR, world record; Values represent the mean percentage difference (and standard deviation, SD) relative to the current WR, accompanied by 95% confidence intervals (CI). Positive values indicate performance above the current WR.*

# Table S5. Current and previous World Records from each sports modality, divided by sex and categories

| Sport | Modality | Category | Sex | Current WR | Previous WR | Difference between  current and past WR (%) |
| --- | --- | --- | --- | --- | --- | --- |
| Track | 100M | NaN | Male | 9.58 | 9.69 | 1.15% |
| Track | 100M | NaN | Female | 10.49 | 10.76 | 2.57% |
| Swimming | 50M | Free | Male | 20.88 | 20.91 | 0.14% |
| Swimming | 50M | Free | Female | 23.61 | 23.67 | 0.25% |
| Swimming | 100M | Free | Male | 46.40 | 46.8 | 0.86% |
| Swimming | 100M | Free | Female | 51.71 | 52.06 | 0.68% |
| Swimming | 50M | Fly | Male | 22.27 | 22.43 | 0.72% |
| Swimming | 100M | Fly | Male | 49.45 | 49.5 | 0.10% |
| Swimming | 50M | Back | Male | 23.55 | 23.71 | 0.68% |
| Swimming | 50M | Breast | Male | 25.95 | 26.42 | 1.81% |
| Swimming | 100M | Breast | Male | 56.88 | 57.1 | 0.39% |
| Weightlifting | Snatch | 88KG | Male | 182.00 | NaN | NaN |
| Weightlifting | Clean&Jerk | 88KG | Male | 220.00 | NaN | NaN |
| Weightlifting | Snatch | 53KG | Female | 99.00 | NaN | NaN |
| Weightlifting | Clean&Jerk | 53KG | Female | 126.00 | NaN | NaN |
| Weightlifting | Snatch | 94KG | Male | 182.00 | NaN | NaN |
| Weightlifting | Clean&Jerk | 94KG | Male | 222.00 | NaN | NaN |
| Weightlifting | Snatch | 86KG | Female | 129.00 | NaN | NaN |
| Weightlifting | Clean&Jerk | 86KG | Female | 162.00 | NaN | NaN |
| Weightlifting | Snatch | 110KG | Male | 196.00 | NaN | NaN |
| Weightlifting | Clean&Jerk | 110KG | Male | 229.00 | NaN | NaN |
| Weightlifting | Snatch | 79KG | Male | 166.00 | NaN | NaN |
| Weightlifting | Clean&Jerk | 79KG | Male | 206.00 | NaN | NaN |
| Weightlifting | Deadlift | NaN | Male | 510.00 | 501 | 1.76% |

*WR, world record; NaN, absent value*

# Table S6. Non-enhanced TEG athletes’ performances differences between TEG and previous PB, stratified by modality, sex, and PB outcome.

| **Subgroup** | **Overall** | **n** |  | **New PB**  **at TEG** | **n** |  | **Non-new PB**  **at TEG** | **n** |
| --- | --- | --- | --- | --- | --- | --- | --- | --- |
| **Overall** | -0.70% ± 1.45%  [-2.22% to 0.82%] | 6 |  | 1.05% ± 0.15%  [-0.27% to 2.37%] | 2 |  | -1.57% ± 0.64%  [-2.60% to -0.55%] | 4 |
| Female | -0.99% ± —  [—] | 1 |  | — | 0 |  | -0.99% ± —  [—] | 1 |
| Male | -0.64% ± 1.61%  [-2.64% to 1.36%] | 5 |  | 1.05% ± 0.15%  [-0.27% to 2.37%] | 2 |  | -1.77% ± 0.62%  [-3.32% to -0.22%] | 3 |
| **Track** | -1.57% ± 0.82%  [-8.97% to 5.83%] | 2 |  | — | 0 |  | -1.57% ± 0.82%  [-8.97% to 5.83%] | 2 |
| Female | -0.99% ± —  [—] | 1 |  | — | 0 |  | -0.99% ± —  [—] | 1 |
| Male | -2.15% ± —  [—] | 1 |  | — | 0 |  | -2.15% ± —  [—] | 1 |
| **Swimming** | -0.26% ± 1.58%  [-2.78% to 2.25%] | 4 |  | 1.05% ± 0.15%  [-0.27% to 2.37%] | 2 |  | -1.58% ± 0.75%  [-8.30% to 5.14%] | 2 |
| Female | — | 0 |  | — | 0 |  | — | 0 |
| Male | -0.26% ± 1.58%  [-2.78% to 2.25%] | 4 |  | 1.05% ± 0.15%  [-0.27% to 2.37%] | 2 |  | -1.58% ± 0.75%  [-8.30% to 5.14%] | 2 |
| **Weightlifting** | — | 0 |  | — | 0 |  | — | 0 |
| Female | — | 0 |  | — | 0 |  | — | 0 |
| Male | — | 0 |  | — | 0 |  | — | 0 |

*TEG, The Enhanced Games; PB, personal best. Values represent the mean percentage difference (and standard deviation, SD) relative to previous PB performance, accompanied by 95% confidence intervals (CI). Positive values indicate performance above previous PB.*

# Table S7. Overall TEG athletes’ performances differences between TEG and previous PB, stratified by modality, sex, and PB outcome.

| **Subgroup** | **Overall** | **n** |  | **New PB**  **at TEG** | **n** |  | **Non-new PB**  **at TEG** | **n** |
| --- | --- | --- | --- | --- | --- | --- | --- | --- |
| **Overall** | -2.66% ± 7.08%  [-4.40% to -0.92%] | 66 |  | 2.90% ± 2.53%  [1.74% to 4.05%] | 21 |  | -5.25% ± 7.04%  [-7.37% to -3.13%] | 45 |
| Female | -2.85% ± 6.23%  [-5.95% to 0.26%] | 18 |  | 4.26% ± 2.34%  [0.54% to 7.99%] | 4 |  | -4.88% ± 5.44%  [-8.02% to -1.74%] | 14 |
| Male | -2.59% ± 7.44%  [-4.75% to -0.43%] | 48 |  | 2.57% ± 2.53%  [1.27% to 3.88%] | 17 |  | -5.42% ± 7.74%  [-8.26% to -2.58%] | 31 |
| **Track** | -5.27% ± 4.99%  [-8.29% to -2.26%] | 13 |  | — | 0 |  | -5.27% ± 4.99%  [-8.29% to -2.26%] | 13 |
| Female | 6.06% ± 7.02%  [-1.31% to 13.42%] | 6 |  | — | 0 |  | 6.06% ± 7.02%  [-1.31% to 13.42%] | 6 |
| Male | 4.60% ± 2.76%  [2.04% to 7.15%] | 7 |  | — | 0 |  | 4.60% ± 2.76%  [2.04% to 7.15%] | 7 |
| **Swimming** | -0.21% ± 2.84%  [-1.17% to 0.75%] | 36 |  | 2.11% ± 2.02%  [1.07% to 3.15%] | 17 |  | -2.29% ± 1.55%  [-3.03% to -1.55%] | 19 |
| Female | 0.26% ± 2.90%  [-2.17% to 2.68%] | 8 |  | 3.26% ± 1.47%  [-0.40% to 6.92%] | 3 |  | -1.54% ± 1.68%  [-3.63% to 0.54%] | 5 |
| Male | -0.34% ± 2.86%  [-1.45% to 0.77%] | 28 |  | 1.86% ± 2.08%  [0.66% to 3.06%] | 14 |  | -2.55% ± 1.48%  [-3.40% to -1.70%] | 14 |
| **Weightlifting** | -5.85% ± 11.73%  [-11.88% to 0.18%] | 17 |  | 6.24% ± 1.60%  [3.69% to 8.79%] | 4 |  | -9.57% ± 10.92%  [-16.17% to -2.97%] | 13 |
| Female | -4.23% ± 8.35%  [-17.52% to 9.05%] | 4 |  | — | 0 |  | -8.07% ± 4.03%  [-18.08% to 1.94%] | 3 |
| Male | -6.35% ± 12.85%  [-14.11% to 1.41%] | 13 |  | 5.89% ± 1.77%  [1.49% to 10.30%] | 3 |  | -10.02% ± 12.43%  [-18.91% to -1.13%] | 10 |

*TEG, The Enhanced Games; PB, personal best. Values represent the mean percentage difference (and standard deviation, SD) relative to previous PB performance, accompanied by 95% confidence intervals (CI). Positive values indicate performance above previous PB.*

# Table S8 – Non-enhanced TEG athletes’ age at previous PB and difference in age between PB and TEG (Δage), stratified by PB achievement status (new PB vs. no new PB).

| **Subgroup** | **New PB performances** | |  |  | **Non-new PB performances** | |  |
| --- | --- | --- | --- | --- | --- | --- | --- |
|  | **Age at PB**  **(years)** | **Δage**  **(years)** | **n** |  | **Age at PB**  **(years)** | **Δage**  **(years)** | **n** |
| **Overall** | 21.00 ± —  [—] | 1.00 ± —  [—] | 2 |  | 23.50 ± 2.52  [19.50 to 27.50] | 3.75 ± 1.26 [1.75 to 5.75] | 4 |
| **Track** | — | — | 0 |  | 25.00 ± 2.83  [-0.41 to 50.41] | 4.50 ± 0.71  [-1.85 to 10.85] | 2 |
| **Swimming** | 21.00 ± —  [—] | 1.00 ± —  [—] | 2 |  | 22.00 ± 1.41  [9.29 to 34.71] | 3.00 ± 1.41  [-9.71 to 15.71] | 2 |
| **Weightlifting** | — | — | 0 |  | — | — | 0 |

*PB, personal best; TEG, The Enhanced Games; Δage, difference in years between age at TEG competition and age at which the previous PB was achieved.*

# Table S9 – Overall TEG athletes’ age at previous PB and difference in age between PB and TEG (Δage), stratified by PB achievement status (new PB vs. no new PB).

| **Subgroup** | **New PB performances** | |  |  | **Non-new PB performances** | |  |
| --- | --- | --- | --- | --- | --- | --- | --- |
|  | **Age at PB**  **(years)** | **Δage**  **(years)** | **n** |  | **Age at PB**  **(years)** | **Δage**  **(years)** | **n** |
| **Overall** | 25.00 ± 4.17  [23.10 to 26.90] | 4.95 ± 3.40 [3.41 to 6.50] | 21 |  | 25.81 ± 4.24  [24.58 to 27.04] | 6.81 ± 3.78 [5.72 to 7.91] | 48 |
| **Track** | — | — |  |  | 26.08 ± 3.25  [24.11 to 28.04] | 4.85 ± 2.88 [3.10 to 6.59] | 13 |
| **Swimming** | 24.18 ± 3.92  [22.16 to 26.19] | 5.47 ± 3.56 [3.64 to 7.30] | 17 |  | 26.11 ± 4.67  [23.86 to 28.35] | 7.37 ± 4.00 [5.44 to 9.30] | 19 |
| **Weightlifting** | 28.50 ± 3.70  [22.62 to 34.38] | 2.75 ± 1.26 [0.75 to 4.75] | 4 |  | 25.25 ± 4.61  [22.79 to 27.71] | 7.75 ± 3.77 [5.74 to 9.76] | 16 |

PB, personal best; TEG, The Enhanced Games; Δage, difference in years between age at TEG competition and age at which the previous PB was achieved.

# Table S10. Non-enhanced TEG athletes’ age at PB and age difference between PB and TEG’s performances, stratified by modality and sex.

| **Subgroup** | **Age at PB**  **(years)** | **Δage**  **(years)** |
| --- | --- | --- |
| **Overall**  **(n = 6)** | 22.67 ± 2.34  [20.21 to 25.12] | 2.83 ± 1.72  [1.03 to 4.64] |
| Females  (n = 1) | 23.00 ± —  [—] | 5.00 ± —  [—] |
| Males  (n = 5) | 22.60 ± 2.61  [19.36 to 25.84] | 2.40 ± 1.52  [0.52 to 4.28] |
| **Track**  **(n = 2)** | 25.00 ± 2.83  [-0.41 to 50.41] | 4.50 ± 0.71 [-1.85 to 10.85] |
| Females  (n = 1) | 23.00 ± —  [—] | 5.00 ± —  [—] |
| Males  (n = 1) | 27.00 ± —  [—] | 4.00 ± —  [—] |
| **Swimming**  **(n = 4)** | 21.50 ± 1.00  [19.91 to 23.09] | 2.00 ± 1.41  [-0.25 to 4.25] |
| Females  (n = 0) | — | — |
| Males  (n = 4) | 21.50 ± 1.00  [19.91 to 23.09] | 2.00 ± 1.41  [-0.25 to 4.25] |
| **Weightlifting**  **(n = 0)** | — | — |
| Females  (n = 0) | — | — |
| Males  (n = 0) | — | — |

PB, personal best; TEG, The Enhanced Games; Δage, difference in years between age at TEG competition and age at which the previous PB was achieved.

# Table S11. Overall TEG athletes’ age at PB and age difference between PB and TEG’s performances, stratified by modality and sex.

| **Subgroup** | **Age at PB**  **(years)** | **Δage**  **(years)** |
| --- | --- | --- |
| **Overall**  **(n = 69)** | 25.57 ± 4.21  [24.55 to 26.58] | 6.25 ± 3.74  [5.35 to 7.15] |
| Females  (n = 20) | 24.85 ± 2.62  [23.62 to 26.08] | 7.35 ± 4.30  [5.34 to 9.36] |
| Males  (n = 49) | 25.86 ± 4.69  [24.51 to 27.21] | 5.80 ± 3.44  [4.81 to 6.78] |
| **Track**  **(n = 13)** | 26.08 ± 3.25  [24.11 to 28.04] | 4.85 ± 2.88  [3.10 to 6.59] |
| Females  (n = 6) | 25.50 ± 2.26  [23.13 to 27.87] | 4.17 ± 0.75  [3.38 to 4.96] |
| Males  (n = 7) | 26.57 ± 4.04  [22.84 to 30.30] | 5.43 ± 3.91  [1.81 to 9.04] |
| **Swimming**  **(n = 36)** | 25.19 ± 4.38  [23.71 to 26.68] | 6.47 ± 3.87  [5.16 to 7.78] |
| Females  (n = 8) | 24.00 ± 1.41  [22.82 to 25.18] | 8.75 ± 3.28  [6.00 to 11.50] |
| Males  (n = 28) | 25.54 ± 4.88  [23.64 to 27.43] | 5.82 ± 3.82  [4.34 to 7.30] |
| **Weightlifting**  **(n = 20)** | 25.90 ± 4.55  [23.77 to 28.03] | 6.75 ± 3.96  [4.90 to 8.60] |
| Females  (n = 6) | 25.33 ± 4.03  [21.10 to 29.57] | 8.67 ± 6.09  [2.28 to 15.06] |
| Males  (n = 14) | 26.14 ± 4.88  [23.32 to 28.96] | 5.93 ± 2.50  [4.49 to 7.37] |

*PB, personal best; TEG, The Enhanced Games; Δage, difference in years between age at TEG competition and age at which the previous PB was achieved.*

# Figure S1. 100m track sprint performance for females and males.

NE, non-enhanced athlete; TEG, The Enhanced Games; PB, personal best performance.

# Figure S2. 50 and 100m Swimming freestyle performance for females and males.

NE, non-enhanced athlete; TEG, The Enhanced Games; PB, personal best performance. Panel A, 50m freestyle performances.; Panel B, 100m freestyle performances.

# Figure S3. Swimming and Weightlifting performances for females and males.

NE, non-enhanced athlete; TEG, The Enhanced Games; PB, personal best performance. Panel A, 50m swimming backstroke performances; Panel B, 50m and 100m swimming breaststroke performances; Panel C, 50m and 100m swimming fly performances; Panel D, deadlift weightlifting performances.

# Figure S4. Correlation between the age and performance differences before and after TEG for non-enhanced athletes.

TEG, The Enhanced Games; y-axis expresses the relative difference between previous PB and TEG performance (positive values indicates increased performance at TEG when compared to PB); x-axis expresses the age difference (in years) between age at TEG and age at previous PB. Slope and intercept accompanied by p-values (between parenthesis) and 95% confidence intervals (between brackets) are presented.

# Figure S5. Correlation between the age and performance differences before and after TEG for all athletes.

TEG, The Enhanced Games; y-axis expresses the relative difference between previous PB and TEG performance (positive values indicates increased performance at TEG when compared to PB); x-axis expresses the age difference (in years) between age at TEG and age at previous PB. Slope and intercept accompanied by p-values (between parenthesis) and 95% confidence intervals (between brackets) are presented.
